# Effects of Hospital Payment Reform of Government Budget Allocation and Social Health Insurance in a Pilot in China

**DOI:** 10.34172/ijhpm.8891

**Published:** 2025-09-09

**Authors:** Ying Meng, Binglun Wu, Liqun Wu, Litian Jiang, Weijia Lu, Huatang Zeng, Jin Xu

**Affiliations:** ^1^School of Public Health, Peking University, Beijing, China.; ^2^China Center for Health Development Studies, Peking University, Beijing, China.; ^3^Shenzhen Health Development Research and Data Management Center, Shenzhen, China.; ^4^Vanke School of Public Health, Tsinghua University, Beijing, China.

**Keywords:** Hospital Efficiency, Hosipital Payment Reform, China

## Abstract

**Background:**

Government budget allocation (GBA) and social health insurance (SHI) constitute the primary revenue sources for public hospitals in China, with GBA accounting for a substantially smaller proportion compared to SHI. Starting in 2015, a megacity in southern China gradually introduced payment reforms. GBA, previously based on government approved number of staff (input-based), was replaced with an output-based model. Subsequently, SHI payment method was changed from fee-for-service (FFS) to case-based payment.

**Methods:**

The study adopted a staggered difference-in-differences (DiD) model to assess the effects of reforms on service volume and capacity, as well as hospital efficiency. We used hospital-level panel data of 29 hospitals in the city from 2009 to 2022.

**Results:**

The GBA reform increased annual outpatient visits by 301 374 per hospital (*P*<.01) and promoted efficiency (score increase of 0.02, *P*<.05). In contrast, the SHI reform increased annual inpatient discharges by 2417 per hospital (*P*<.05) but had no significant effect on efficiency (-0.15, *P*>.1).

**Conclusion:**

The output-based GBA reform increased outpatient service volume and the number of healthcare professionals, while the case-based SHI reform raised inpatient discharges. Only GBA was associated with a modest efficiency gain given the distinct incentives of GBA and SHI. Future research should explore strategies for better alignment of multiple funding streams, such as unified purchasing with blended payment models or clearer functional differentiation.

## Background

**Key Messages**

**Implications for policy makers**

- This study represents the first comprehensive examination of the effects of payment reforms on multiple funding streams of hospitals in China. It provides significant empirical evidence from a developing country context, where hospitals commonly depend on diverse funding sources. The findings provide valuable insights into how financial incentives can be leveraged to influence hospital behaviour.
- Our study finds that both the government budget allocation (GBA) and social health insurance (SHI) reforms significantly increased service volumes, with GBA linked to higher outpatient and emergency visits, and SHI to more inpatient discharges. The GBA reform was further associated with a modest efficiency gain and a significant rise in healthcare professionals. Although GBA accounted for a relatively small share of hospital revenue, it had a considerable impact on hospital behavior.
- The two public hospital funding streams, namely GBA and SHI payments, are managed by different agencies and utilize distinct payment methods. Although both finance healthcare service delivery, they differ in structure and may generate conflicting incentives. To reduce administrative costs and improve the efficiency of fund utilization, it is essential to harmonize these funding streams and align their objectives.

**Implications for the public**

Our study investigated the effects of payment reforms on hospital behaviours in China, where hospitals operate with multiple funding streams. The analysis revealed that both government budget allocation (GBA) and social health insurance (SHI) significantly increased hospital service volume; however, only the government budget reform resulted in an increase in healthcare professionals and improved efficiency. Financial incentives demonstrated the most substantial impact on hospital output. Moreover, given the rapid expansion of hospital scale during the study period, the reforms had only limited effects on efficiency. These findings offer valuable empirical insights for other countries with similar funding structures that are considering hospital payment reforms.

Public hospitals serve as the dominant providers of healthcare services in China.^1^ Improving hospital efficiency through reform is considered critical in achieving universal health coverage in the country.^2^ Unlike many countries, where often no clear distinction exists between social health insurance (SHI) and direct government spending, public hospitals in China rely on both service charges (about 88% of hospital revenue in 2019, covered by SHI and out-of-pocket spending) and direct government spending (about 10% of hospital revenue). Direct government spending for hospital services (referred to as government budget allocation, or GBA), accounts for about half of overall direct government spending on public hospitals.^3^ While the payment model for service charges has traditionally been fee-for-service (FFS), under which providers are paid for each service performed, the GBA has been based on a government approved quota for personnel numbers or bed counts (input-based). Hospitals were allowed to hire additional healthcare professionals beyond the personnel quota, based on their own operational needs. The FFS payment for SHI has caused concerns of over commercialization,^4^ while GBA payment based on inputs was considered to provide little incentive to improve service efficiency.^5^

 Although both serve as compensation for healthcare services, GBA and SHI remain independent funding streams in China, governed by different agencies and utilizing distinct payment methods. This not only increases administrative costs but also creates potential discrepancies in financial incentives. Debate persists over whether to increase GBA as a distinct funding source or to merge it with SHI.^3^ Recent policies related to public hospital payment reforms in China promoted both output-based GBA payment and case-based SHI payment reforms, in order to improve the efficient use of healthcare resources. Under the output-based model, hospitals are reimbursed based on the volume of services provided. Meanwhile, under the case-based payment system, providers receive for each admission a fixed amount specific to each case group.^6^

 Recent literature reviews on payment for public hospitals have generally focused on moving from FFS to case-based payment (sometimes using the term “activity-based payment”), and consistently found that case-based payment reforms tended to reduce the length of stay, while the effects on efficiency were mixed.^7-10^ However, the distinct two-stream public funding model for public hospitals in China means these results may be partially relevant at best, and evidence from China is needed to inform public hospital payments reform in China. Yet evidence is highly limited on both case-based SHI reform and output-based GBA reform. Evaluation of a policy change from FFS to case-based payment in Beijing and Guangzhou reported no significant changes in hospital efficiency or admission volumes,^11,12^ but we failed to find any study in English on reform of the Chinese GBA payment model.

 A megacity in southern China has since 2015 uniquely launched a GBA payment reform that replaced the input-based model with an output-based model, and a SHI payment reform that replaced FFS with case-based payment. It provides a rare opportunity to observe the effects of almost simultaneous reforms on both revenue streams of public hospitals. In light of the agency theory, there exists inherent information asymmetry and potential goal divergence between the government (as principal) and hospitals (as agents).^13^ Through payment method reforms, government could incentivize hospitals to act in line with the direction it establishes. Under the reformed GBA, which has shifted from an input- to an output-based model, hospitals are incentivized to increase service volume—either by expanding capacity or improving efficiency. Meanwhile, the shift of SHI from FFS to case-based payment for inpatient care, encouraging hospitals to increase inpatient admissions and shorten lengths of stay, which might also affect capacity or operational efficiency.

 Utilizing hospital-level data from the city of interest covering the period 2009-2022, this study aimed to evaluate the impact of payment reform on the service volume, service capacity and efficiency of public hospitals. To the best of our knowledge, this represents the first empirical analysis to provide critical evidence on the GBA payment reform, and also the first investigation into hospital payments reforms that concurrently address two distinct funding streams and payment models.

## Methods

###  Study Design and Setting 

 The GBA reform was implemented in the city we studied in phases from 2015 to 2020, while the SHI reform was implemented in phases from 2019 to 2021. We used a staggered difference-in-differences (DiD) approach to compare outcomes between hospitals that implemented reforms early and those yet to reform. Besides, we conducted event studies to analyse how reform effect evolved over time and to examine parallel trend assumptions of hospital payment reform.

 The city of interest had a total of 17.79 million residents in 2023. The city’s proportion of the population aged 65 or above was 3.22% in 2020, well below the national average (13.5%),^14^ but increased nearly doubled since 2010 (1.76%), outpacing the speed of ageing nationwide. Second, the city has a highly developed economy, with a per capita gross domestic product of almost 200 000 in Chinese Yuan in 2023 (28 278 in 2023 US dollars), making it one of the richest cities in China. Amid the rapid expanding numbers of hospital beds all around China, the number of hospital beds in the city increased by 221% from 2009 to 2023.

 In order to improve the hospital efficiency to meet the growing demand of healthcare, the city replaced the input-based model of GBA payment with an output-based one starting from the beginning of 2015. The new payment mechanism introduced a flat tariff paid per year retrospectively for each outpatient and emergency visit and each inpatient day, with no adjustment for patient type or complexity. It should be noted that, while the personnel quota was also lifted along with GBA reform, there was no restriction on the total number of healthcare professionals, either before or after the reform. On the other hand, hospital bed numbers continued to be stringently regulated. The tariffs were calculated in such a way as to minimize changes in the total subsidy to hospitals, in order to facilitate a smooth policy transition. Furthermore, since 2019, the conventional FFS model of SHI payment for hospitalizations was shifted to case-based payment, where patients were categorized into groups based on the complexity of their condition and the resources required for their treatment. Hospitals were then reimbursed based on these case groups, irrespective of the actual costs incurred during treatment.

 As the SHI reform coincided with the COVID-19 outbreak, we could not entirely eliminate the possibility that the pandemic confounded our estimates of the reform’s effects. Apart from the pandemic, neither a review of policy documents nor informal interviews with hospital administrators and policy-makers identified any concurrent heterogeneous policies that could bias the estimated effects.

###  Data and Variables

####  Data

 We obtained hospital-year data for 2009 to 2022 from the local Health Statistics Report. Ths report follows a standardized national template and protocols for data collection, verification, and submission, under the supervision of national health authorities. These procedures ensure high data quality and reliability. We restricted the sample to public hospitals operating continuously throughout the study period. To ensure homogeneity among studied samples, we included only hospitals with a minimum of 100 beds (eligibility criteria for accreditation as a secondary or tertiary hospital). We also excluded one infectious disease hospital with atypical data due to the COVID-19 pandemic, and one hospital that implemented GBA reform after SHI reform, as this reversed the typical sequence and could confound results. The final sample included 29 public hospitals that adopted the SHI payment reform subsequent to the GBA reform. Figure S1 (Supplementary file 1) shows the varying implementation timelines across hospitals in introducing the payment reforms from 2009 to 2022.

####  Outcome Variables

 Outcome variables we studied as service volume indicators included outpatient and emergency visits, discharged inpatients, and total inpatient days per year. We also used the number of healthcare professionals and beds in each year to represent service capacity. We excluded financial inputs due to data limitations. Finally, the efficiency score for each hospital year was calculated using data envelopment analysis (DEA), with outpatient and emergency visits, as well as discharged inpatients, as the output vector and healthcare professionals and beds as the input vector.^15-17^

 DEA constructed the efficiency frontier, representing the optimal combinations of outputs and inputs, where maximal output was achieved at a given level of input, or minimal input was achieved at a given level of output, through linear programming.^18-20^ Each hospital was compared to the frontier, yielding a relative efficiency score, with higher values indicating greater efficiency. To ensure hospitals from different periods are evaluated against the same frontier, we used the Malmquist global frontiers model, which constructed a single frontier using data from all time periods. These efficiency scores were analysed using MaxDEA software (version X 12.1).

###  Statistical Analysis

 We used staggered DiD model proposed by Callaway and Sant’Anna (CS-DiD),^18^ which was considered suitable for the varied time points of policy implementation in the city. Specifically, we ran two sets of CS-DiD models, one for the GBA payment reform and the other for SHI payment reform. In the first set of models, the GBA reform was specified as the independent variable, and we excluded observations following the SHI reform to estimate the net effects of the GBA reform. In contrast, the second model treated the SHI reform as the independent variable. We put those observations back in but excluded observations before the GBA reform to estimate the net effects of the SHI reform on those that had already gone through GBA reform.

 As explained in Callaway and Sant’Anna,^18^ in instances where the timing of treatment differs across groups and the effects of treatment change over the duration of the treatment, traditional two-way fixed effect (TWFE) models may yield biased estimates of treatment effects that lean towards zero.^24^ This phenomenon is attributed to the potential presence of heterogeneous policy effects, thereby suggesting that estimations obtained through the TWFE model may be comparatively understated relative to those obtained via the CS-DiD model. We therefore used CS-DiD to address the issue of varying time of introduction of reform.

 Under the CS-DiD framework, we first stratified hospitals into distinct groups based on their respective reform implementation timings. We then estimated group-specific treatment effects τ(g,t) for each treatment group (g) in post-implementation periods (t) using a 2 × 2 DiD design. This design automatically eliminates individual-fixed effects and time-fixed effects through differencing computation, with the identification and estimation of effects relying on individual-level change and then averaging to the group level.^18^

 We conducted an event study to test the pre-reform trend of outcomes in both the treatment and control groups. In addition, to test the robustness of our findings, we performed a series of sensitivity analyses, including a placebo test using alternative treatment timing and a TWFE DiD model. The results of these robustness checks are presented in Supplementary file 2.

## Results

###  Summary Statistics

Table 1 presents the characteristics of sample hospitals by hospital-year observations before both GBP and SHI reforms, after GBA reform and before SHI reform, and after both reforms. The data in Table 1 highlights marked increase in service delivery and capacity across hospitals after each round of reform. However, the average efficiency score experienced a slight decrease, from 0.89 before the reforms to 0.86 after the GBA reform, and further to 0.84 after both reforms. Notably, service capacity expanded substantially with number of beds increased from 491 to 979, and number of healthcare professionals increased from 924 to 1728.

**Table 1:** Key Characteristics of the Sample Hospitals Grouped by Status of Reform, 2009-2022 (Mean, 95% Confidence Interval) (Per Hospital Per Year)

	**Variables**	**Before Both GBP and SHI ** **(N = 224)**	**After GBP and Before SHI ** **(N = 112)**	**After Both GBP and SHI ** **(N = 70)**
Service volume	Outpatient and emergency visits	1 377 413 (1 262 986 to 1 491 840)	1 550 979 (1 373 233 to 1 728 724)	1 842 810 (1 606 286 to 2 079 335)
	Inpatient discharges	19 257 (173 267 to 21 188)	28 116 (23 767 to 32 465)	38 448 (31 072 to 45 823)
	Total inpatient days	153 905 (136 751 to 171 059)	218 128 (184 085 to 252 172)	274 222 (222 911 to 325 533)
Service capacity	Beds	491 (443 to 539)	734 (636 to 832)	979 (824 to 1133)
	Healthcare professionals	924 (850 to 997)	1247 (1109 to 1384)	1728 (1483 to 1972)
Efficiency	Efficiency score	0.89 (0.88 to 0.90)	0.86 (0.84 to 0.87)	0.84 (0.82 to 0.87)

Abbreviations: GBA, Government budget allocation; SHI, social health insurance.

###  Impact on Service Volume

**Figure 1:**
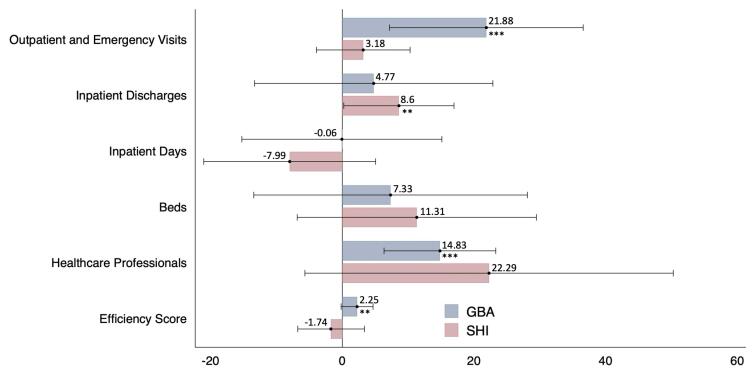
Effects of Two Reforms as a Percentage of Pre-reform Baselines (%) (Per Hospital Per Year). Abbreviations: GBA, Government budget allocation; SHI, social health insurance.

Table 2 presents the results of CS-DiD models. The public hospital payment reforms significantly boosted service volumes. Specifically, the GBA reform significantly increased outpatient and emergency visits by 301 374 per hospital annually (*P* < .01), representing 21.88% of the average prior to both the GBA and SHI reforms (See Figure 1). It had no significant effects on annual per-hospital inpatient discharges (919, *P* > .1) or inpatient days (-92, *P* > .1). Conversely, the SHI reform led to a notable rise in inpatient discharges, with an annual increase of 2417 discharges per hospital (*P* < .05), accounting for 8.6% of the average level prior to SHI reform. It did not significantly influence either outpatient and emergency visits (49 383, *P* > .1) or the total number of inpatient days (-17 426, *P* > .1).

**Table 2:** Impact of Hospital Payment Reform on Annual Service Volume, Service Capacity and Efficiency of Public Hospitals (2009-2020)^a^ (Per Hospital Per Year)

	**Service Volume**			**Service Capacity**		**Efficiency**
	**Outpatient and Emergency Visits**	**Inpatient Discharges**	**Inpatient Days**	**Beds**	**Healthcare Professionals**	**Efficiency Score**
GBA (N = 313)	301 374^***^ (103 402)	919 (1778)	-92 (11 931)	36 (52)	137^***^ (40)	0.02^**^ (0.011)
SHI (N = 122)	49 383 (56 295)	2417^**^ (1201)	-17 426 (14 525)	83 (68)	278 (178)	-0.15 (0.022)

Abbreviations: GBA, Government budget allocation; SHI, social health insurance. ^a^ In the analysis of the GBA reform effect, the years 2020 to 2022 were automatically omitted because all sample hospitals had implemented GBA during these years, thus eliminating the control group. Similarly, the years 2021 to 2022 were automatically omitted in the analysis of SHI reform effect. ^*^ *P* <.1, ^**^ *P* <.05, ^***^ *P* <.01.

###  Impact on Service Capacity 

 According to Table 2, the GBA reform was associated with a non-significant annual increase of 36 beds per hospital (*P* > .1) and a statistically significant annual increase of 137 healthcare professionals (*P* < .01), representing 14.83% of the average prior to both the GBA and SHI reforms. The SHI reform was associated with a non-significant annual increase of 83 beds (*P* > .1) and 278 healthcare professionals (*P* > .1) per hospital.

###  Impact on Efficiency 

 The GBA reform demonstrated a modest, yet statistically significant improvement in efficiency with a score increase of 0.02 (*P* < .05) per hospital per year, representing 2.25% of the average prior to both the GBA and SHI reforms. In contrast, The SHI reform showed no significant impact on hospital efficiency, with a coefficient change of -0.15 (*P* > .1).

###  Event Study

**Figure 2:**
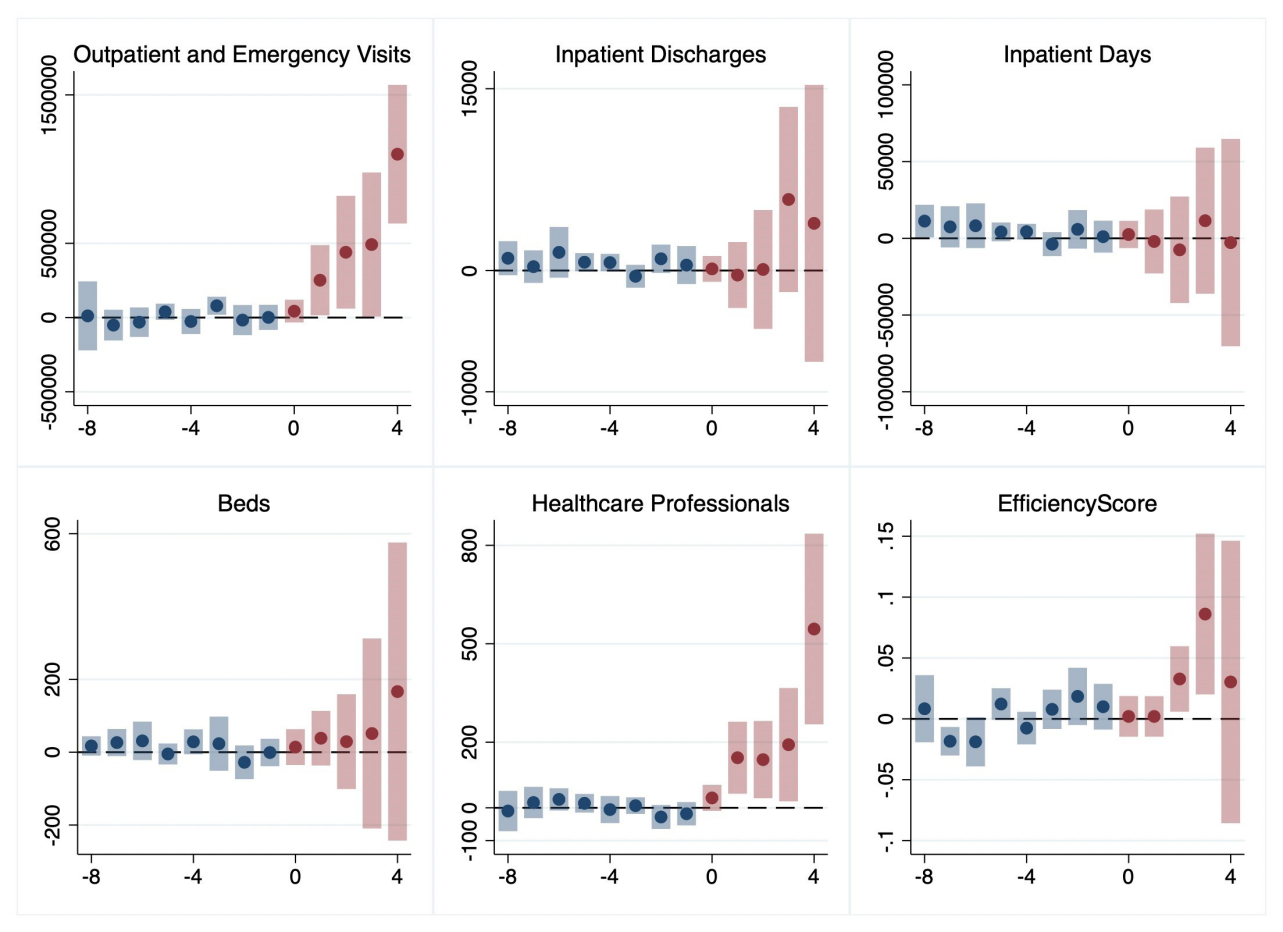
Government Budget Allocation Reform Event Study Estimates (Per Hospital Per Year).

**Figure 3:**
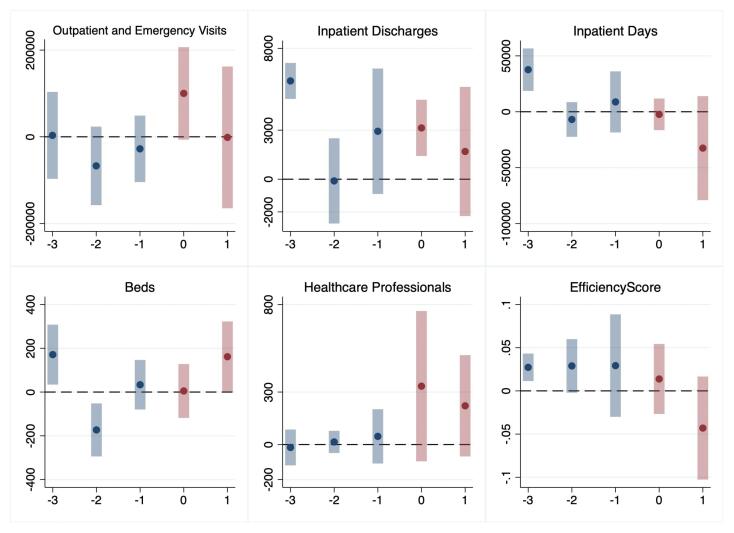
Social Health Insurance Reform Event Study Estimates (Per Hospital Per Year).

Figures 2 and 3 display point estimates and corresponding 95% confidence intervals for the average impact of the reform on selected metrics in public hospitals. Each data point represents the reform’s estimated effect on a specific outcome within a specified timeframe. Year 0 marks the first year of the reform implementation. From Figure 2, we can see that there were no obvious pre-existing trends in the primary outcomes before the sequential reforms. On the other hand, due to the scarcity of pre-implementation observations of SHI reform, particularly from three years prior, the assumption of parallel trends was less robustly established than that of the GBA payment reform. Meanwhile, there was no other notable reform between GBA and SHI reforms among sample hospitals, which along with lack of substantial pre-trend differences supported the conclusion that the effects identified were attributable to the reforms.

 The post-reform effects are consistent with the findings of the CS-DiD model. Following the GBA reform, there was an increase in the number of outpatient and emergency visits, the number of healthcare professionals, and the efficiency score. In contrast, after the SHI reform, the number of inpatient discharges increased. It should be noted that, as the years progressed, the number of observations after the reform decreased, and this reduction in observations might have contributed to the gradually increasing effects observed in the GBA model.

## Discussion

 This study evaluates a comprehensive payment reform involving both GBA and SHI funding streams for public hospitals. It is the first paper to assess the impact of shifting GBA from a traditional input-based model to an output-based model in China, and also the first to evaluate a two-step reform in which both GBA and SHI payments were restructured, with the latter transitioning from FFS to case-based payment. We find an increase in outpatient and emergency visits under GBA reform, and an increase in inpatient discharges under SHI payment reform. We also observe an increase in efficiency under the GBA payment reform, but our results do not provide evidence of a statistically significant efficiency change following the SHI reform. Additionally, only GBA payment reform was associated with expansion in hospital capacity, evident from the increased number of healthcare professionals, an outcome that was not the stated intention of the reform.

 While much of the existing literature on hospital payment reform in China has focused primarily on SHI, this study examines both GBA and SHI reforms. This dual focus reflects the common reality in many low- and middle-income countries (LMICs), where hospitals are financed through multiple funding streams.^19-21^ Existing studies on multiple funding streams are largely qualitative, however, our causal evidence shows that even a minor funding stream can affect hospital behavior.

 The GBA payment reform significantly increased outpatient and emergency visits variables, however, it did not show a significant effect on either inpatient discharges or inpatient days, despite both being directly tied to the payment calculation base. The discrepancy between outpatient and inpatient care is unsurprising, given that unmet needs for outpatient services in China were higher and grew faster between 2008 and 2018, while those for inpatient care remained stable.^22^ The per capita volume of outpatient visits in China remained below the Organisation for Economic Co-operation and Development (OECD) average.^23^ Therefore, it was likely easier to increase volume of outpatient care than inpatient admissions (and therefore discharges). Additionally, the public hospital performance evaluation system that favored shorter lengths of stay,^24^ might have counterbalanced the incentive to increase bed days introduced by the output-based GBA reform. Furthermore, the increased outpatient visits might have also partially replaced the need for long hospital stays.

 The fact that GBA reform led to an increase in the number of healthcare professionals but not beds, likely because the number of healthcare professionals was not restricted either before or after the removal of quota (used to calculate amount of GBA), while bed numbers are tightly regulated. The increase in staff size without parallel increase in beds also fit with the outpatient-focused service growth and efficiency gain observed in our study. Lastly, although the absolute efficiency gain from the GBA reform may seem small (0.02), the magnitude of this efficiency imporvement is considerable when compared with the narrow range of efficiency score changes (0.05) and standard deviations (0.11).

 Our findings on the effects of SHI payment reform are in line with studies regarding increased inpatient service utilization following the implementation of case-based payment from Gemany,^25^ England,^26^ Korea, China, and Thailand.^27^ However, our study shows no significant effect of case-based payment reform on outpatient service volume contrasting findings from Korea^28^ and Switzerland.^29^ A previous systematic review showed that shifting from FFS to case-based payment reform was overall associated with lowered cost and shortened length of stay,^27^ but our results reveal no significant efficiency gains from the case-based payment. This discrepancy may arise because our analysis used hospital-level data and the DEA method to assess efficiency, whereas other studies typically employ case-level or diagnosis-group-level data to assess changes in the average length of stay, serving as a proxy for hospital efficiency. DEA is considered advantageous as a method to analyse hospital efficiency as it uses a comprehensive measure of multiple inputs and outputs, rather than a single or arbitrarily selected indicator(s).^30^

###  Policy Implications

 Several implications can be drawn from our study. First, our findings suggest that output-based payments are more effective than input-based payments in incentivizing public hospitals to increase service volume and efficiency. As input-based payment remains prevalent for GBA to most hospitals in China, our study provides empirical evidence to support the adoption of output-based reforms in other settings.

 Second, harmonization of funding streams and payment mechanisms is likely beneficial in strengthening strategic purchasing in China and similar settings in LMICs where multiple funding streams are frequently found. The partially conflicting incentives presented by GBA (rewarding service expansion) and SHI (eg, promoting shorter stays) highlight the need for a harmonized financing system, potentially through a blended mechanism that combines multiple payment methods.^31^ Interdepartmental fragmentation may hinder such harmonization. A unified or coordinated purchasing mechanism, such as merging GBA and SHI management or creating a joint purchasing body, could address this.^32^ Alternatively, GBA could fund hospital-based public health services to fill prevention financing gaps.^33^ The optimal approach for harmonizing funding streams should be tailored to local contexts in China and other LMICs.^34^

###  Limitation and Future Research

 This study is subject to several limitations. Firstly, the DEA model did not include financial inputs; however, this limitation is partly mitigated by the fact that financial inputs in Chinese public hospitals are largely composed of labor costs, which overlap with the labor inputs already captured in our model. Second, without data on case-mix, this limitation may lead to an overestimation or underestimation of efficiency for hospitals with significant variations in case-mix, thereby affecting the results regarding the reform’s impact on efficiency. Thirdly, we were unable to explore the likely spillover effects of the hospital payment reform on primary healthcare institutions due to data restrictions; increased hospital visits could mean more bypassing of primary care facilities and inefficient allocation of resources. Fourthly, support for the parallel trends assumption required in the CS-DiD models assessing the SHI payment reform was restricted by the small number of pre-implementation observations. In addition, although the estimation methods employed in our study are widely applied in empirical economics research, the COVID-19 outbreak and certain unobservable factors might still influence the policy effect estimates. Finally, the study is restricted to one (albeit major) city, which may imply limited statistical generalizability of our findings. However, the study has theoretical generalizability, as coexistence of separate GBA (based on input) and SHI payment is generally consistent across the country, while the reform is also well aligned with the overall guidelines of hospital payment reform in China, which involves both GBA and SHI payments for hospital services.

 Future studies could incorporate more comprehensive data, including non-labor financial inputs, quality of care, and case-mix characteristics, to enhance the comprehensiveness of assessments. It is important to investigate whether gains in efficiency and staffing are associated with enhancements in care quality and reductions in per-admission or per-visit costs. Replicating the analysis in other cities and over a longer period would improve understanding of context sensitivity and allow a more robust, generalizable evaluation of the payment reforms’ long-term effects. Furthermore, future studies should explore how to better align multiple funding streams across different health system contexts, either through a unified or coordinated purchasing mechanism or a clearer functional differentiation.

## Conclusion

 The public hospital payment reforms implemented between 2015 and 2021 had a significant impact on public hospitals. The output-based GBA reform resulted in an expansion of both outpatient service volume and healthcare professionals, while the case-based SHI reform boosted inpatient discharges. However, the increase in inpatient discharges under the SHI reform did not translate into efficiency gains, with only the GBA reform demonstrating a modest positive effect on hospital efficiency. The two streams of GBA and SHI create distinct financial incentives. Future research should explore how to better align them, either through a unified or coordinated purchasing mechanism with blended payment models, or a clearer functional differentiation.

## Acknowledgements

 We thank Professors Anne Mills and Timothy Powell-Jackson for their critical comments on drafts of the manuscript. We alone remain responsible for the content of the paper and any errors are our own. We also thank Yi Wei for conducting data cleaning in the preliminary phase of the study.

## Ethical issues

 We utilized administrative data from hospitals, which did not involve personal data. According to the requirements of Peking University, ethics approval was not necessary.

## Conflicts of interest

 Authors declare that they have no conflicts of interest.

## Supplementary files

Supplementary file 1. The Progress of Hospital Payment Reforms.

Supplementary file 2. Sensitivity Analysis.
