# Supplementary material for: Effects of Hospital Payment Reform of Government Budget Allocation and Social Health Insurance in a Pilot in China
Source: Int J Health Policy Manag. 2025 Sep 9;14:8891. doi: 10.34172/ijhpm.8891 (PMC12595573; doi:10.34172/ijhpm.8891)

Article title: Effects of Hospital Payment Reform of Government Budget Allocation and Social Health Insurance in a Pilot in China

Journal name: International Journal of Health Policy and Management (IJHPM)

Authors' information: Ying Meng[1,2], Binglun Wu[3], Liqun Wu[3], Litian Jiang[3], Weijia Lu[1,2], Huatang Zeng[3,4]*, Jin Xu[2]*

[1]School of Public Health, Peking University, Beijing, China.

[2]China Center for Health Development Studies, Peking University, Beijing, China.

[3]Shenzhen Health Development Research and Data Management Center, Shenzhen, China.

[4]Vanke School of Public Health, Tsinghua University, Beijing, China.

***Correspondence to:** Huatang Zeng; Email: zht22@tsinghua.edu.cn & Jin Xu; Email: xujin@hsc.pku.edu.cn

Citation: Meng Y, Wu B, Wu L, et al. Effects of hospital payment reform of government budget allocation and social health insurance in a pilot in China. Int J Health Policy Manag. 2025;14:8891. doi:10.34172/ijhpm.8891

Supplementary file 1. The Progress of Hospital Payment Reforms

Figure S1 depicts the trajectory of payment reform implementation across 29 hospitals in the city of interest from 2009 to 2022. The vertical axis identifies the hospitals by their codes (H1-H29), and the horizontal axis represents the timeline of the study. The figure utilizes a gradient of grey to indicate the reform status of each hospital: white signifies no reforms initiated, light grey indicates completion of the GBA reform, and dark grey indicates that the hospital has completed both reforms.

Figure S1. The progress of hospital payment reforms, 2009-2022

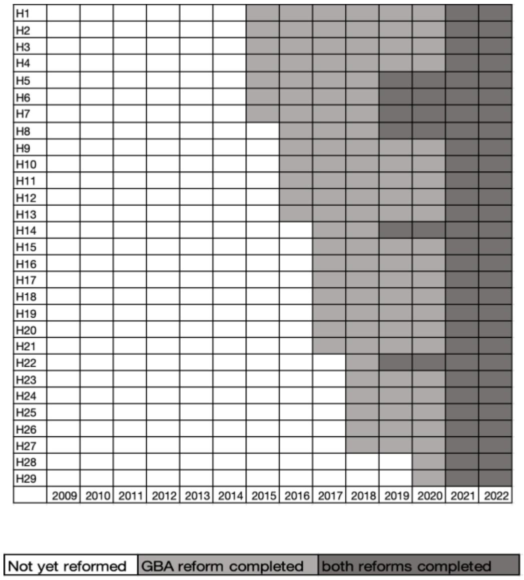

Supplement: Supplementary file 1 — The Progress of Hospital Payment Reforms. [file ijhpm-14-8891-s001.pdf]
