# Supplementary material for: Effects of Hospital Payment Reform of Government Budget Allocation and Social Health Insurance in a Pilot in China
Source: Int J Health Policy Manag. 2025 Sep 9;14:8891. doi: 10.34172/ijhpm.8891 (PMC12595573; doi:10.34172/ijhpm.8891)
Supplement: Supplementary file 2 — Sensitivity Analysis. [file ijhpm-14-8891-s002.pdf]

Article title: Effects of Hospital Payment Reform of Government Budget Allocation and Social Health Insurance in a Pilot in China

Journal name: International Journal of Health Policy and Management (IJHPM)

Authors' information: Ying Meng[1,2], Binglun Wu[3], Liqun Wu[3], Litian Jiang[3], Weijia Lu[1,2], Huatang Zeng[3,4]*, Jin Xu[2]*

[1]School of Public Health, Peking University, Beijing, China.

[2]China Center for Health Development Studies, Peking University, Beijing, China.

[3]Shenzhen Health Development Research and Data Management Center, Shenzhen, China.

[4]Vanke School of Public Health, Tsinghua University, Beijing, China.

***Correspondence to:** Huatang Zeng; Email: zht22@tsinghua.edu.cn & Jin Xu; Email: xujin@hsc.pku.edu.cn

Citation: Meng Y, Wu B, Wu L, et al. Effects of hospital payment reform of government budget allocation and social health insurance in a pilot in China. Int J Health Policy Manag. 2025;14:8891. doi:10.34172/ijhpm.8891

Supplementary file 2. Sensitivity Analysis

aTable 1 presents the results of the placebo test using alternative treatment timing. To conduct this test, we first systematically shifted the reform implementation date forward by one year to simulate hypothetical treatment scenarios. Second, we restricted all placebo analyses to the pre-reform period to avoid contamination from actual policy effects during the post-reform phase. This conservative design helps ensure the internal validity of our placebo test.

The placebo test shows no statistically significant effects for both GBA and SHI groups under fake treatment timing (TableS1), supporting the validity of the actual policy effects.

Table S1. Placebo test using alternative treatment timing (per hospital per year)

Variables	Service volume			Service capacity		Efficiency
	Outpatient and Emergency Visits	Inpatient Discharges	Inpatient Days	Beds	Healthcare Professionals	Efficiency Score
GBA (N=220)	1420	453	1078	-1	-18	0.010
	(43241)	(800)	(5306)	(19)	(18)	(0.010)
SHI (N=62)	-27754	2933	8792	33	47	0.029
	(39107)	(1958)	(13944)	(58)	(79)	(0.030)

* p < 0.1, ** p < 0.05, *** p < 0.01
[a]In the analysis of the GBA reform effect, the years 2020 to 2022 were automatically omitted because all sample hospitals had implemented GBA during these years, thus eliminating the control group. Similarly, the years 2021 to 2022 were automatically omitted in the analysis of SHI reform effect.

Table S2 reports the results of the TWFE DiD model. Unlike the CS-DiD estimates, the TWFE model finds significant effects of the GBA reform on inpatient discharges, inpatient days, and the number of beds, while the SHI reform remains non-significant across all outcomes. This difference likely stems from the TWFE model's assumption of homogeneous treatment effects across groups and time. When treatment effects are heterogeneous, this assumption may lead to biased estimates. Overall, the CS-DiD results appear more conservative and robust in our research context.

Table S2. Impact of hospital payment reform on annual service volume, service capacity and efficiency of public hospitals (2009-2020)[a] (per hospital per year) (TWFE DiD model)

Variables	Service volume			Service capacity		Efficiency
	Outpatient and Emergency Visits	Inpatient Discharges	Inpatient Days	Beds	Healthcare Professionals	Efficiency Score
GBA (N=336)	142839***	2630***	18460***	64**	59*	0.024***
	(62525)	(1092)	(8417)	(35)	(33)	(0.008)
SHI (N=182)	-15134 (60652)	-1529 (1962)	-21468 (14177)	-39 (47)	193 (103)*	-0.010 (.0190)

* p < 0.1, ** p < 0.05, *** p < 0.01
[a]In the analysis of the GBA reform effect, the years 2020 to 2022 were automatically omitted because all sample hospitals had implemented GBA during these years, thus eliminating the control group. Similarly, the years 2021 to 2022 were automatically omitted in the analysis of SHI reform effect.
